# Evaluation of Different Wastewater Treatment Processes and Development of a Modified Attached Growth Bioreactor as a Decentralized Approach for Small Communities

**DOI:** 10.1155/2013/156870

**Published:** 2013-11-12

**Authors:** Shohreh Azizi, Alireza Valipour, Thami Sithebe

**Affiliations:** ^1^Department of Biological Sciences, Faculty of Agriculture, Science and Technology, North-West University, Mafikeng Campus, Private Bag X2046, Mmabatho 2735, South Africa; ^2^Department of Civil Engineering, Yeungnam University, Gyungsan 712-749, Republic of Korea

## Abstract

This study was undertaken to evaluate the potential future use of three biological processes in order to designate the most desired solution for on-site treatment of wastewater from residential complexes, that is, conventional activated sludge process (CASP), moving-bed biofilm reactor (MBBR), and packed-bed biofilm reactor (PBBR). Hydraulic retention time (HRT) of 6, 3, and 2 h can be achieved in CASP, MBBR, and PBBR, respectively. The PBBR dealt with a particular arrangement to prevent the restriction of oxygen transfer efficiency into the thick biofilms. The laboratory scale result revealed that the overall reduction of 87% COD, 92% BOD_5_, 82% TSS, 79% NH_3_-N, 43% PO_4_-P, 95% MPN, and 97% TVC at a HRT of 2 h was achieved in PBBR. The microflora present in the system was also estimated through the isolation, identification, and immobilization of the microorganisms with an index of COD elimination. The number of bacterial species examined on the nutrient agar medium was 22 and five bacterial species were documented to degrade the organic pollutants by reducing COD by more than 43%. This study illustrated that the present PBBR with a specific modified internal arrangement could be an ideal practice for promoting sustainable decentralization and therefore providing a low wastage sludge biomass concentration.

## 1. Introduction

The primary renewable source of freshwater is rainfall, which generates a global supply of 40 000–45 000 km^3^ per year [1]. This more or less constant water supply must support the entire world population, which is increasing at a constant rate [2]. Thus, the per capita accessibility of freshwater is decreasing at a very fast rate. During the last few decades, the number of countries experiencing water scarcity has increased. Apart from the scarcity of freshwater in many countries, the developing countries in particular, the quality of the available freshwater is also deteriorating due to pollution, hence intensifying the shortage. Liquid wastes such as untreated sewage or industrial waste are the major sources of pollutants in developing countries. Wastewater reuse is an important approach for conservation of water resources, particularly in areas suffering from water shortage. However, wastewater treatment plants represent one of the major investments due to high capital cost in addition to operation and maintenance cost. In developing countries lack of funding results in inadequate operation of wastewater treatment plants [3]. Moreover, big residential complexes with high population densities can be served by decentralized systems that are simpler and cost effective. With growing population and rapid urbanization, the land availability has become scare and setting up centralized sewage treatment plant is not a viable option. The large capital investment of sewerage system and pumping costs associated with centralized systems can be reduced, thus increasing the affordability of wastewater management systems.

The aerobic processes are known biological practices involved in treating domestic wastewater and offer an on-site effective solution in areas with low population densities, especially in sectors of residential complexes [4]. The efficiency of processes primarily depends on the biomass concentration and specific conversion rate of the microorganisms [5]. Over the past few decades the biological efforts were generally based on two distinct principles of suspended growth and attached growth routes [6].

The conventional activated sludge process is a suspended growth technology comprising of an enrichment culture of microbial consortia in order to remove impurities and transform wastewater into environmentally acceptable quality [7]. In this system the culture is retained to maintain convenient sludge age and treatment reaction rates. The microorganisms absorb organic material to grow and form the flocs of biomass [8, 9]. However, the attached growth systems are advanced to the suspended biomass processes. Attached growth creates the biofilm on the support media to provide a better treatment efficiency due to accumulation of high microbial population in the presence of large surface area [10, 11]. The shape and size of biomass-supporting media can also play a significant role in the design of biofilm processes in order to meet an obligatory surface area for microbial growth [12]. The microorganisms secrete a sort of natural polymer to facilitate firm adhesion on inert support matrix for biofilm development and biooxidation mechanism [13, 14]. Numerous investigations have demonstrated the efficiency of the attached growth unit processes in wastewater treatment, although the key advantage of these practices is rarely exploited in full-scale processes due to oxygen transfer limitations into thick biofilms [15]. In that order, the packed-bed biofilm technologies have high specific surface area and fixed biomass concentration leading to a smaller volume of reactor, while biofiltration techniques may cause choking and clogging dilemma [16, 17]. Likewise, the moving-bed biofilm reactor is incorporated with the advantage of conventional activated sludge and fixed-film practices [11, 18]. Thus, it is significantly important for overcoming some of the apparent limitations and evaluate the performance of biological systems where the most suitable technologies are available for on-site residential wastewater treatment. The comparative research also could lead to knowledge sharing of appropriate selection and operation of treatment techniques, particularly in developing countries [19].

The present scientific approach is an attempt to compare and review the potential future use of three aerobic biological systems, namely, conventional activated sludge process (CASP), moving bed biofilm reactor (MBBR), and packed-bed biofilm reactor (PBBR) for on-site treatment of wastewater from residential complexes. The packed-bed biofilm reactor is operated under a modified specific arrangement to improve the performance of the process, reduce the limitations of attached growth technologies, and create a particular air distribution pattern for possible oxygen penetration into thick biofilms. The microbiological studies were also performed to examine and document the bacterial cells which have potential for the degradation of organic pollutants. 

## 2. Materials and Methods

### 2.1. Pilot Plant Setup

**Figure 1:**
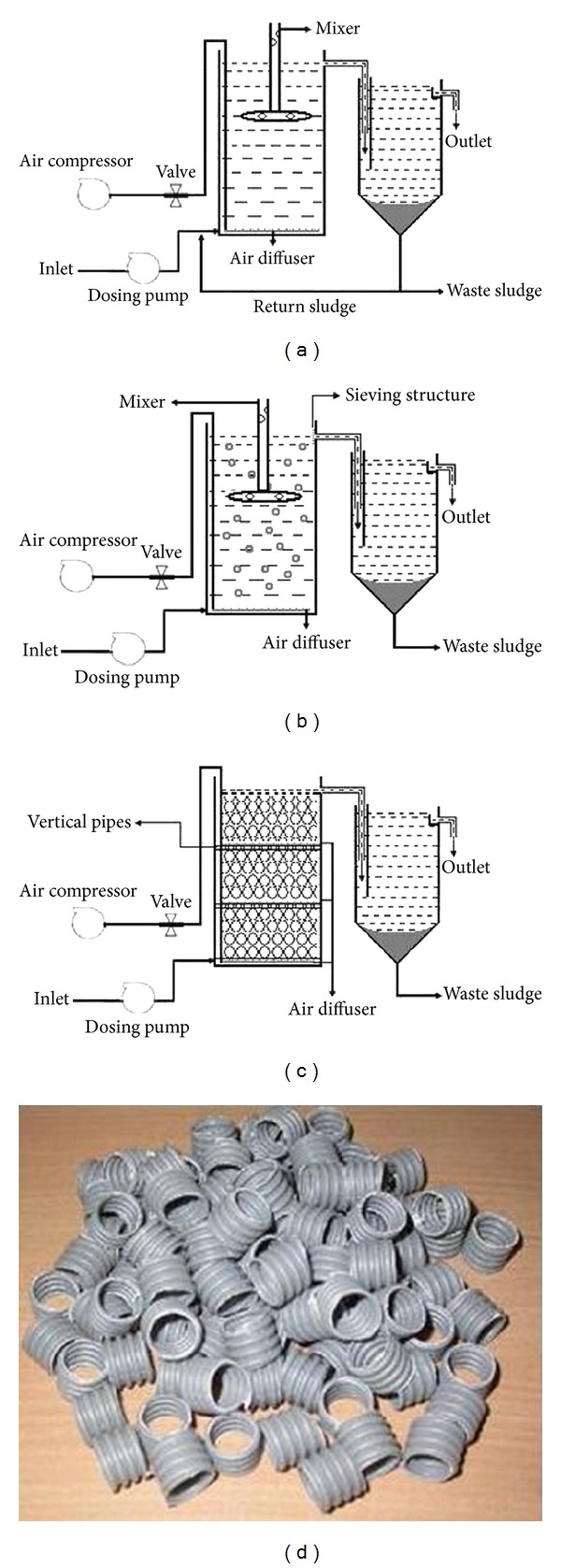
The schematic appearance of biological treatment processes ((a) ASP, (b) MBBR, (c) PBBR, (d) carrier media).

The three laboratory units were used to evaluate the performance of three biological processes for residential wastewater treatment, that is, conventional activated sludge process (CASP), moving bed biofilm reactor (MBBR), and modified-packed bed biofilm reactor (PBBR). These three systems have the same size and dimension (Table 1). The schematics of the pilot plants are given in Figure 1. The conventional activated sludge process was operated as per standard practices [15]. The experiments were conducted by using both aerator and mixer. The mixer arm had a perforated hole which was blowing the air to supply into the reactor. The flow rate was maintained by peristaltic pump as well as constant head box in all the three systems (Figure 1(a)). The mesh aperture size of 2 to 6 mm was used to manually screen the raw wastewater before entering into the storage feed tank. The screened effluent was discharged into the reactor by a standard dosing pump to degrade the organic matters under aerobic condition. The MBBR had a cylindrical shaped polypropylene carrier media to support biofilm growth (Figure 1(b)). The unit consists of a main bioreactor and a settler. The effective depth of the reactor was 320 mm filled with plastic packing carrier. Filling ratio of packing carrier in the reactor is important due to the amount of biomass which can be supported by carriers. The requirement of volume of carrier media (v/v) was optimized during the experimentation. The main feature in the PBBR system is the arrangement of fixed bed in layered strata as indicated in Figure 1(c). Between the layered strata a vertical pipe arrangement was made for ease of effluent flow. This configuration avoids choking of sludge and for air distribution different header pipes to various levels were provided for uniform distribution of air in the reactor. Such configuration increases the oxygen transfer efficiency in each layer compared to MBBR where the bottom air supply is available for the entire reactor. The void ratio of the reactor was calculated to be 92.18%. Controlled sewage was fed at the bottom of the reactor keeping sufficient upflow velocity to prevent clogging. An air compressor was used to supply the air required for the reactor and injected from bottom. The reactor was packed with a lid; there are some holes on it for flowing atmospheric air through it. The upper and lower part of media were fixed with mesh in each layer. The effective volume of the reactor was approximately 10 liters in which the media were submerged. The media in PBBR was similar to that used in MBBR and had the same surface area and characteristics (Figure 1(d)).

### 2.2. Sampling and Analysis

The domestic wastewater samples were collected from the residential complexes on a daily basis to carry out a series of extensive experiments for the duration of 245 days. Samples were collected from the inlet and outlet of reactors every day to analyze the temperature, pH, chemical oxygen demand (COD), biochemical oxygen demand (BOD_5_), total suspended solids (TSS), ammonium nitrogen (NH_3_-N), phosphate (PO_4_-P), and most probable number (MPN) of coliform bacteria as per the standard methods. The most probable number (MPN) of the carrier media is measured by scraping the surface of the media in known volume of wastewater sample, and then the sample was taken for MPN through the standard method of estimation [20]. The analytical values are the mean of five replicates. The performance evaluation was done based on the effluent discharge norms specified by the local pollution control board. The excess sludge of biological processes was examined for the parameters as suspended solids (SS), volatile suspended solids (VSS), and sludge volume index (SVI) through the standard practices [15, 20]. The biomass inside the carrier media is measured in terms of MLSS and estimated by using known volume of media containing the biofilm. The particle size analysis of waste sludge was carried out using the laser diffraction method (Malvern Mastersizer 2000, UK). The samples of inlet and outlet effluents, biofilm media surfaces, and mixed liquor of the conventional activated sludge process were practiced for total viable count (TVC) of bacterial population, isolated on nutrient agar medium, and identified. The Gram-negative bacterial identification was done using Mini API (bioMérieux SA, France), and for Gram-positive, Bergey's Manual of Determinative Bacteriology [21] was used. The immobilization and ability studies of microbial cells on the percentage degradation of chemical oxygen demand (COD) were examined by inoculating isolated bacteria to the flask containing 100 mL sterile wastewater and incubated at 37°C with 180 rpm for 24 h and the wastewater was sterilized at 121°C for 20 min. 

The active attached biomass on the surface of carrier media was observed by using a Stereoscan 440 scanning electron microscope (SEM, Leica, Cambridge, UK) as per the standard procedure [22]. 

## 3. Results and Discussion

### 3.1. Characteristic of Wastewater from Residential Complex

The composition of domestic wastewater varies with time and rate of water used and depends upon the life quality, living habits, culture, climatic conditions, community size, and developmental level. However, the residential complex concentration of organic pollutant is higher than the municipal sewage due to low dilution and high organic load (high concentration of residential use). The high organic load is due to discharge of kitchen waste containing oil and waste containing detergents. The characteristics of raw sewage from residentinal complex and sewage municipality wastewater are shown in Table 2.

### 3.2. Start-Up and Acclimatization

In the activated sludge process the biomass seeding was done using an active sludge obtained from a sewage treatment plant. The biosolid concentration was 3000 mg in 50 mL of volume. The initial MLSS concentration was 280 mg/L which reached to 3000 mg/L after 20 days. A low and controlled effluent flow was fed to the reactor for the generation of higher biomass and acclimatization. The continuous feed was slowly increased from 25% feed flow to reaching 100% of flow rate over a period of 15 days. The hydraulic retention time was kept at 14 h at 100% feed rate. Periodically outlet water was monitored for COD and BOD removal till a constant quality was obtained and after 20 days sludge build-up was recorded to be 3000 mg/L.

After packing the reactor with the carrier media in MBBR and PBBR, 3 liters of sludge from the returned sludge line of an activated sludge system from treatment plant was added to both the reactors in order to provide the initial microbial mass. Then, 7 liters of domestic wastewater was added to the reactor. The hydraulic regime of the reactor was slowly increased from 30% flow reaching up to 100% flow rate over a period of 25 days and the hydraulic retention time (HRT) was adjusted at 14 h. After the complete establishment of biomass on the carriers media and achievement of steady state conditions of BOD and COD concentration of the pilot plant effluent, the data of 245 consecutive days was analyzed to calculate optimum hydraulic retention time values.

### 3.3. Optimization of the Carrier Media in MBBR

Optimization of media is a critical factor to attain effective treatment efficiency and also effective microbial growth. The percentage of carrier media in the reactor is governed by the volume of reactor and can be limited to 70% [23]. However, the percentage of media required is based on wastewater characteristics and specific treatment goals. Adequate turbulence is ideal for efficient system performance. 

The organic loading rate is governed by the media fill ratio (v/v) in the reactor. Fill ratio is normally indicated by space occupied by media in the reactor volume. On the basis of fill ratio ranging from 20% to 30%, 40%, 50%, and 60% of the reactor the optimization studies were carried out at 6 h of hydraulic retention time. It was noted that 40% of media are optimum for effective treatment, while with increasing media fill ratio, the COD reduction was almost constant. The organic loading rate at 40% carrier media was 0.024 kg/m^2^/m^3^ of surface area considering the organic load to be approximately 600 mg/L COD and the active surface area to be 350 m^2^/m^3^. Increasing the surface area of media percentage does not make any change due to constant organic loading. However, decreasing the area below 40% results in significant reduction in the COD removal efficiency due to availability of less surface area for microbial biofilm.

### 3.4. Performance of the Reactors

The experimental assays were conducted to estimate the optimum hydraulic retention time (HRT) for effective elimination of impurities. Under steady state condition the biological systems were operated between 12 and 1 h HRT with overall average BOD_5_ loading between 6 and 80 g/d approximately in CASP system. The organic loading per unit surface area of the media was within the ranges of 5.28–60.11 and 2.20–23.47 g/m^2^/d in MBBR and PBBR. The processes were exceeded to run at each retention time over a period of 25 days after achieving effluent characteristics with constant concentration. 

**Figure 2:**
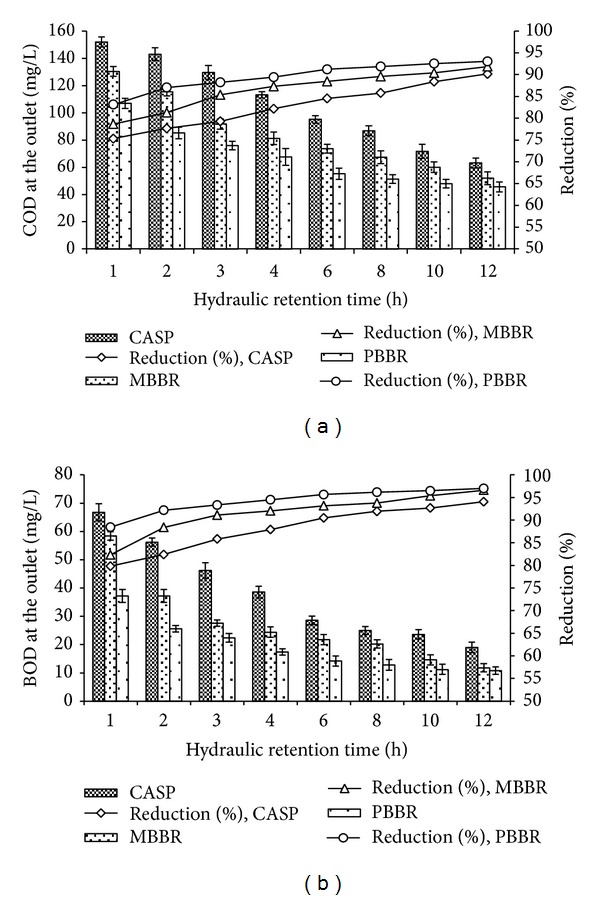
Average COD (a) and BOD_5_ (b) values of treated effluents through the biological processes. The values represent the mean of five replicates ± standard error (SE).

The performance of the systems showed that COD and BOD_5_ concentrations of treated effluent were below 100 and 30 mg/L at above 6, 3, and 2 h HRT in CASP, MBBR, and PBBR, respectively. Thus, the hydraulic retention times of 6, 3, and 2 h were considered optimal for achieving adequate organic removal from residential wastewater under given operating and designing conditions (Figure 2). Thus in contrary to conventional activated sludge process the biofilm based systems have high treatment efficiency and reaction rate. This is primarily because of the greater biomass accumulation and high microbial growth. The specific modified arrangement of the present packed bed biofilm reactor is intended to overcome the possible restriction of oxygen transfer efficiency and provides a relatively greater effluent quality at a higher organic loading rate. The high oxygen transfer efficiency might have led to reduction of the power consumption as stated by relevant literature on methods for enabling energy conservation (USEPA, 1999; USEPA, 2010; UTA, 2010).

The optimum HRT of 2 h in biological packed bed system to some extent was also found to be lower than reported for attached growth processes [12, 24]. The CASP and MBBR have shown identical operating conditions [15, 25].

### 3.5. Overall Treatment Performance

The comprehensive evaluation of the reduction percentage of other parameters was conducted at optimum operating conditions to assess the performance of the biological treatment systems (Table 3). The results confirmed that the attached growth biological reactors provided a reliable means of elimination of pollutants by lower retention time at the outlet end of the processes. The packed bed biofilm treatment unit presents more valuable rate of purification owning to absolute metabolism and synthesis of degradable organic matters with enriched active biomass concentration. The pH value ranged between 7 and 8 at the treated effluent of the three purification practices.

The packed bed biofilm reactor in contrast to other biological systems confirms to accomplish a considerably better performance of organic degradation. It offers the advantage of lower wastage of biomass concentration as it facilitates its greater management (Table 4).

The conventional activated sludge process was found to be responsible for 3.48 × 10^7^ cfu/mL of bacterial consortia in the reactor (MLSS: 3000 mg/L). The carrier biofilm media provide evidence of enormous TVC concentration with an average of 3.74 × 10^7^ cfu/cm^2^ by means of 8000–9000 mg/L solids. The presence of greater surface area per unit volume enhances the bacterial population responsible for organic degradation through the PBBR process [26].

Table 3 also showed improvement in the performance of PBBR compared to other systems. This is primarily because of the specific arrangement of fixed bed provided with low pressure drops through the beds and reduced channeling through the beds. This led to an improved hydraulic residence time distribution which has significant effects in terms of quality control of out-flowing treated water and in terms of potential reduction in reactor size and sludge volume and cost. The present configuration reduced the channeling phenomena and effectively reduced surface area which helped a better contact with the polluted water and therefore effective biodegradation was facilitated. This also contributed to the presence of extra oxygenation and helped the sludge in getting mineralized. The mineralization results in lesser biomass at the outlet of the system and reduced the menace of handling sludge. Thus lower organic load would then require lower hydraulic retention time and even 1.0 h of HRT is also achievable [27] but the type and strength of organic wastewater in domestic wastewater will vary the HRT for organic degradation.

### 3.6. Microbial Investigation

**Figure 3:**
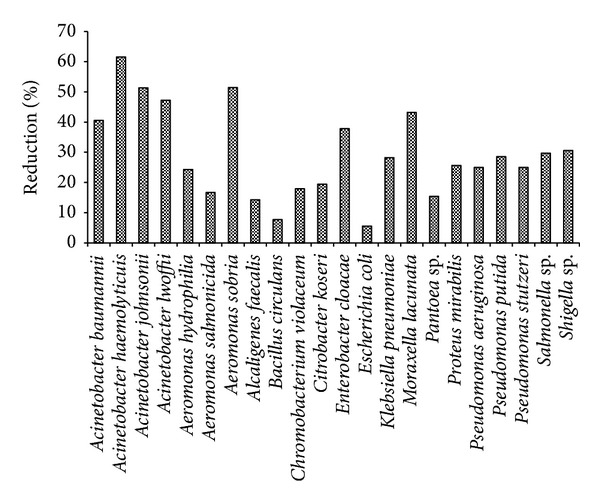
The microbial communities and their profiles in terms of average percentage removal of chemical oxygen demand (COD).

**Figure 4:**
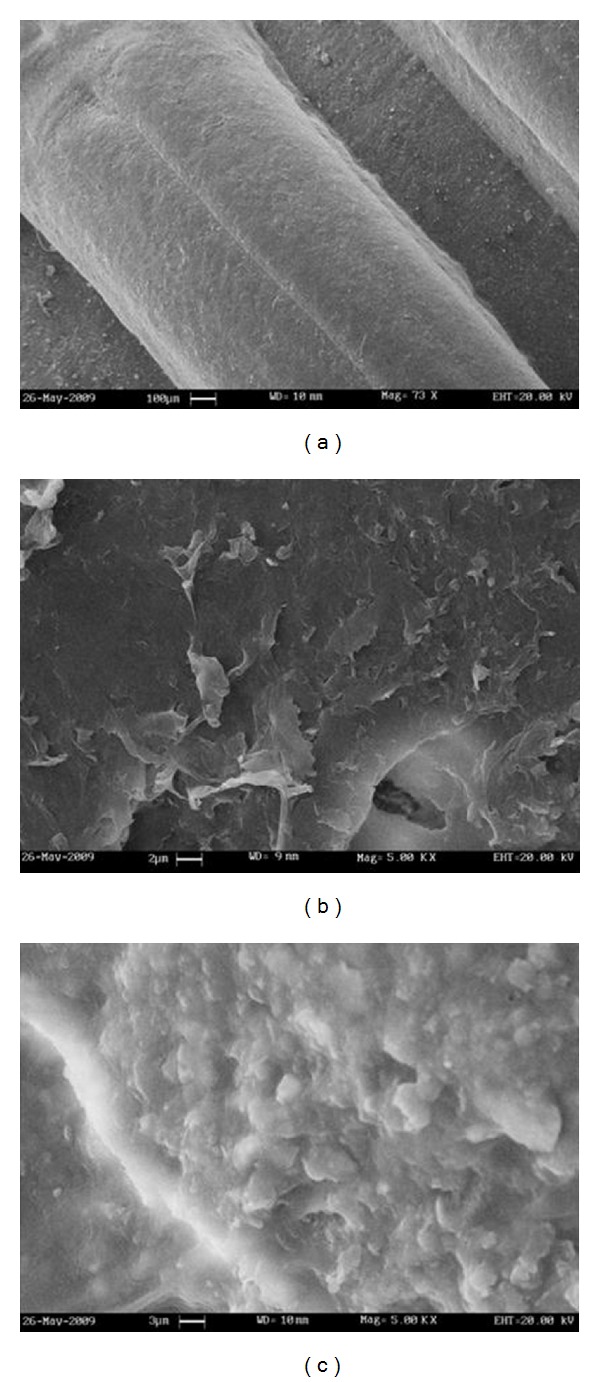
SEM photographs of the biofilm-forming bacteria on the surface of the carrier media before treatment at 73x magnification (a) and after treatment at 5 kx magnification: MBBR (b) and PBBR (c).

The species of microorganism which frequently dominate in any biological system depend on the environmental conditions, process design, and plant operation [28]. The isolation, and the identification of a microbial consortium were conducted to have an overview of reactions that can occur during the treatment of residential wastewater (Figure 3). The complete profiling of microflora population at various zones of biological processes, that is, inlet, outlet, and aeration tank, and on the biocarrier surface revealed the presence of approximately 22 bacterial strains grown on nutrient agar medium. These bacterial populations were reduced significantly at the outlet of the reactors,which is evident from the results on TVC (Table 3). There is a possibility of the existence of other microbial populations which could not be detected in a nutrient agar medium. Correspondingly, the bacterial immobilization showed the predominance of five microorganisms (*Acinetobacter haemolyticus, Acinetobacter johnsonii, Acinetobacter lwoffii, Aeromonas sobria*, and *Moraxella lacunata*) for the effective degradation of organic pollutants with respect to reduction of COD percentage. A control-immobilized sample showed no significant influence on the COD removal efficiency in the absence of bacterial species. The sticky layers of biofilm as a well-organized community of bacteria are more resilient to process disturbances and they can be considerably more robust, especially when compared to the conventional activated sludge process [29, 30]. For this reason, the biofilm adhesion in the context of bacterial formation was visualized by scanning electron microscope (SEM). The microscopic examinations revealed the bulk densities of biomass concentration on the carrier surface media in both attached growth systems (Figure 4). These biofilms as surface-associated communities of bacteria could play a great role in a biooxidation of organic pollutants, especially in the PBBR by means of large surface area for biomass accumulation. It should be recognized that the large effluent flow at a lower hydraulic retention time can control slime layer deposition on the carrier media through the reactor [15]. The specific arrangement of the packed bed biofilm reactor may considerably offer the framework to overcome certain limitations of percolating bed bioreactors in terms of choking, clogging, oxygen transfer limitation, treatment efficiency diminution, and odor dilemma caused by bacterial activity under anaerobic condition.

## 4. Conclusion

The following conclusions can be drawn from the study.

- The results obtained from the study suggest that the conventional activated sludge has low degree of flexibility and treatment efficiency; however, the attached growth technologies are remarkably superior in pollutant elimination even at low hydraulic retention time from residential wastewater.
- The present packed bed biofilm reactor under modified internal arrangement provided a better treatment efficiency and lower wastage of biosolids in comparison to the other two processes.
- Therefore, this PBBR may create an effective tool to eliminate the disadvantages of choking, clogging, channeling, and so forth. The low HRT can confirm the inexpensive overall capital cost in attached growth processes wherein the land restriction is a vital commodity. The profiling of bacterial isolation, identification, and immobilization indicated five associated microflora in the degradation of organic pollutants based on COD reduction.
- The general pathway of studies shows that the application of present packed bed biofilm reactor could serve as an ideal process and holds a great promise for on-site residential wastewater treatment; however, this calls for detailed pilot scale and field studies.

## Figures and Tables

**Table 1:** The dimensional details and media features of the bench scale system.

Features	Details
Reactor configuration	
Area of the reactor (m^2^)	0.035
Height of the reactor (mm)	320
Volume of the bioreactor (L)	11
Void volume in presence of intervals (L)	10
Settler volume (L)	2.68

Media feature	
Material	Polypropylene
Density (g/cm^3^)	0.95
Shape	Corrugated cylinder
Length (mm)	10
Diameter (mm)	14
Specific surface area (m^2^/m^3^)	350
Fill ratio (%)	
MBBR	40
PBBR	100

**Table 2:** The comparison of the characteristics of residential wastewater and municipal sewage wastewater.

Parameters	Locate	
	Residential	Municipal sewage
Temperature °C	28.86 ± 0.6	27.5 ± 0.68
pH	7.10 ± 0.26	7.12 ± 0.12
COD (mg/L)	632 ± 29.15	420.3 ± 52.02
BOD_5_ (mg/L)	324.03 ± 17	234.5 ± 22.30
TSS (mg/L)	226.20 ± 22.10	157.4 ± 10.50
NH_3_-N (mg/L)	38.50 ± 2.03	32.24 ± 3.72
PO_4_-P (mg/L)	10.48 ± 0.80	8.37 ± 0.57
MPN/100 mL	5.66 × 10^6^ ± 1.7 × 10^6^	5.26 × 10^6^ ± 1.58 × 10^6^
TVC (cfu/mL)	2.91 × 10^7^ ± 5.04 × 10^6^	1.04 × 10^7^ ± 7.38 × 10^6^

The values represent the mean of five replicates ± standard error (SE).

**Table 3:** Summary of the average percentage removal of pollutants from the biological treatment processes at optimum operating conditions.

Parameters	Samples	CASP (6 h HRT)		MBBR (3 h HRT)		PBBR (2 h HRT)	
		Values	Reduction (%)	Values	Reduction (%)	Values	Reduction (%)
TSS (mg/L)	Inlet	207.6 ± 11.15	76.78	233.4 ± 14.60	80.03	251.2 ± 10.25	82.08
	Outlet	48.2 ± 1.30		46.6 ± 1.14		45 ± 1.58	

NH_3_-N (mg/L)	Inlet	36.92 ± 2.05	73.83	38.36 ± 1.76	77.50	40.48 ± 1.61	79.03
	Outlet	9.66 ± 0.28		8.63 ± 0.53		8.49 ± 0.44	

PO_4_-P (mg/L)	Inlet	10.38 ± 0.83	35.64	10.55 ± 0.64	40.28	10.62 ± 0.67	43.31
	Outlet	6.68 ± 0.54		6.30 ± 0.35		6.02 ± 0.38	

MPN/100 mL	Inlet	6.06 × 10^6^ ± 2.7 × 10^6^	94.25	5.56 × 10^6^ ± 1.70 × 10^6^	93.27	6.62 × 10^6^ ± 1.95 × 10^6^	95.44
	Outlet	3.48 × 10^5^ ± 1.43 × 10^5^		3.74 × 10^5^ ± 1.17 × 10^5^		3.02 × 10^5^ ± 1.12 × 10^5^	

TVC (cfu/mL)	Inlet	2.23 × 10^7^ ± 4.49 × 10^6^	95.30	2.88 × 10^7^ ± 3.36 × 10^6^	96.04	3.34 × 10^7^ ± 3.17 × 10^6^	97.16
	Outlet	1.05 × 10^6^ ± 2.71 × 10^5^		1.14 × 10^6^ ± 7.16 × 10^4^		9.48 × 10^5^ ± 6.98 × 10^4^	

The values represent the mean of five replicates ± standard error (SE).

**Table 4:** The average waste sludge characteristics of different aerobic treatment systems.

Parameters	CASP	MBBR	PBBR
SS (mg/L)	5080 ± 286	3240 ± 216	2568 ± 128
VSS (mg/L)	3556 ± 234	1450 ± 112	1130 ± 82
SVI (g/mL)	138 ± 18	76 ± 14	62 ± 10
Particle size (*μ*m)	40–550	25–130	5–50

The values represent the mean of five replicates ± standard error (SE).
